# Pharmacology podcasts: a qualitative study of non-medical prescribing students' use, perceptions and impact on learning

**DOI:** 10.1186/1472-6920-11-2

**Published:** 2011-01-11

**Authors:** Oonagh Meade, Dianne Bowskill, Joanne S Lymn

**Affiliations:** 1 School of Nursing, Midwifery & Physiotherapy, University of Nottingham, Queens Medical Centre, Nottingham, U.K

## Abstract

**Background:**

There is growing research on student use of podcasts in academic settings. However, there is little in-depth research focusing on student experience of podcasts, in particular in terms of barriers to, and facilitators of, podcast use and students' perceptions of the usefulness of podcasts as learning tools. This study aimed to explore the experiences of non-medical prescribing students who had access to podcasts of key pharmacology lectures as supplementary learning tools to their existing course materials.

**Methods:**

Semi-structured interviews were carried out with seven non-medical prescribing students (average age = 43 years), all of whom were nurses, who had access to seven podcasts of key pharmacology lectures. These podcasts took the form of downloadable audio lecture recordings available through the virtual learning environment WebCT. Low, medium and high users of the podcasts took part in the interviews in order to access a variety of student experiences. Interview data was analysed using thematic template analysis to identify key themes surrounding student experience of podcast availability, particularly in relation to barriers to and facilitators of podcast use, and students' experiences of podcasts as a learning tool.

**Results:**

Students used podcasts for a variety of reasons such as revisiting lectures, preparing for exams, to clarify or revise specific topics and, to a lesser extent, to catch up on a missed lecture. Barriers to podcast use centred mainly around technological issues. Lack of experience of the technology required to access podcasts proved a barrier for some students. A lack of access to suitable technology was also a reported barrier. Family assistance and I.T. assistance from the university helped facilitate students' use of the podcasts. Students found that using podcasts allowed them to have greater control over their learning and to gauge their learning needs, as well as helping them build their understanding of a complex topic.

**Conclusions:**

Students used podcasts for a variety of reasons. Barriers to podcasts use were generally related to technological issues. Students often found that once assistance had been gained regarding these technological issues, they accessed the podcasts more easily. Students felt that access to podcasts added value to their learning materials by allowing them to better manage their learning and build their understanding. Podcasts represent a valuable additional learning tool for this specific group of older students.

## Background

Podcasts, or audio recordings of lecture material which can be accessed through the internet or downloaded to a portable media player, have been suggested to be 'an educational revolution in the making' [1]. The ability of these tools to provide truly mobile learning has been recognised by a number of sources [2-4]. Despite this initial excitement there are only limited studies exploring the use of this technology within nursing and medical education [5-11] and several of these have been conducted outside the UK [5,9-11].

Further complicating the literature around the use of podcasts is the uncertainty that exists regarding what the technology is, and how it is being used. A podcast is often defined as an online audio media file which is made available through web syndication [12-14]. However, in educational settings, podcasts (or lecture recordings) are often made available to students via on-line virtual learning environments [3,5,8,10,11,15-17]. Podcasts can also be enhanced by linking the audio recording to PowerPoint slide presentations [5,6,9-11] or through the use of video podcasts, in which case they are known as vodcasts [7,15,18].

A number of studies have utilised podcasting as an alternative to face-to-face lectures [7,10,11] while others have utilised this technology solely for revision exercises [6] and yet others, have utilised podcasts as supplementary learning tools [5,8,9,15-18]. Moreover, in relation to the studies which specifically used podcasts as supplementary learning tools, there is some variation in how podcasts of full lectures are used. Some studies have evaluated the use of audio and video recordings [15,18], while others focus on the evaluation of audio recordings which have been linked with PowerPoint slides from the related lecture [5,9]. Our particular approach to podcasting in non-medical prescribing (NMP) education has been to provide students with access to audio recordings of key pharmacology lectures. These podcasts were housed in the online virtual learning environment WebCT. The podcasts did not replace lectures, but were available to supplement learning. Students had access to either full lecture recordings, or sections of the full-lecture recordings which corresponded with the content of specific slides on their lecture handouts.

Overall, the student view of podcasting in nursing and medical education has been positive with students reporting a beneficial impact on their learning [5-11] and a reduction in anxiety [5]. This is not to say, however, that the introduction of podcasts is without its problems. Students from studies where podcasts have replaced lectures have reported that the inability to ask questions and receive immediate feedback is a disadvantage [7]. There have also been reports of conflicting data in relation to the effect of podcast introduction on the level of student absenteeism from lectures where the introduction of this technology has been used as a supplementary learning tool [8,9,11].

While students have often self-reported in questionnaire studies that podcasts have had a positive impact on their knowledge [5-11], there is limited literature available which has examined the impact of the introduction of podcasts on objective measures of student knowledge and understanding. Vogt et al. [10] conducted an evaluation of the use of podcasts as replacements for traditional lectures across a variety of subjects in undergraduate nursing education. The authors found no significant differences in correct responses to exam questions between a group of students who had access to traditional lectures and a group of students who had access to podcasts of lecture recordings rather than traditional lectures.

Our previous study regarding the use of audio pharmacology podcasts within NMP education is the first to clearly provide objective evidence of the value of these tools in enhancing student knowledge and understanding [8]. Our study differed from Vogt et al. [10] in that podcasts of lectures were made available to students in addition to the traditional lectures. A comparison of exam results between students who had access to the podcasts as additional resources and historical cohorts of students who did not have this additional resource, revealed a significant improvement in understanding of pharmacological concepts among students who had access to the podcasts [8]. As part of our previous study, student perceptions of podcasts were assessed and their use of podcasts objectively tracked. Results suggested that podcasts were highly accessed with students indicating that they found podcasts useful in a number of ways and would like more of these tools introduced across the NMP curriculum [8]. Despite the potential for 'mobile learning' offered by the podcasts, students in our previous study [8] and indeed in other student groups including computer and marine science [15,18] have reported that they generally listen to podcasts through a computer rather than utilising them in the 'mobile' manner intended (i.e. downloading MP3 files to a mobile device). Although there is some similarity across students groups in terms of their mode of accessing podcasts, it is useful to consider at this point the nature of the NMP course and the uniqueness of this particular student population as this will likely impact on student use of podcast material.

Non-medical prescribing in the U.K. is a six month, part-time course which aims to train a range of qualified health care professionals, including nurses, pharmacists, podiatrists, physiotherapists and radiographers, to prescribe drugs. Indeed, following successful completion of the course, these healthcare professionals have access to almost the same formulary of drugs as do doctors [19]. It is critical then that these students develop a good understanding of the principles of pharmacology and therapeutics [20]. This is particularly complicated for nurses attending NMP courses as the move away from a biological to a social model of care at pre-registration level [21] often means that these students do not have the necessary scientific background, exhibited by other professional groups, on which to build this new knowledge. A recent study of nurses enrolling on the NMP course at the University of Nottingham suggested that 50% had no more than a GCSE level understanding of biological science [22]. Indeed, pharmacology has been identified as being an area of particular weakness in nursing education by a number of authors [23-26]. The difficulties for these students are further complicated by both the part-time nature of the course which runs alongside their normal working commitments and the older average age of the student population [8]. The particular problems associated with these sorts of student groups, in terms of juggling family, financial and professional commitments have been well documented [27-29]. While the increased responsibility and accountability in terms of patient safety conferred by a NMP qualification is well recognised by these students [30,31], the requisite development of pharmacological understanding can be a significant challenge.

Our previous study of podcast use with NMP students reported an improvement in objectively-assessed student knowledge, as well as positive student evaluations in questionnaire feedback [8]. Therefore, it is likely that podcasts may play an important role in developing these students' knowledge base. In order to more fully understand the potential value of podcasts as a supplementary learning tool, however, a more in-depth, detailed understanding of students' experiences of using podcasts is required.

By developing a clearer understanding of NMP students' experiences, educators can work to improve student access to these tools and further enhance the development of student understanding in this area. Moreover, this knowledge may be useful for educators interested in developing podcast use across other subjects and disciplines.

The aim of the current study was therefore to develop a detailed qualitative account of how and why students accessed NMP podcasts, their perceptions of the value of podcasts for their learning and to understand more fully any potential barriers towards, and facilitators of, podcast use.

## Methods

### Design

A qualitative design was adopted, using semi-structured interviews to explore student experiences of using podcasts.

### Participants

Participants (n = 7) were recruited from a cohort of NMP students (n = 30) at the University of Nottingham. The majority of students in this cohort were nurses (n = 28) and the remaining two students were pharmacists. Due to the more comprehensive pre-registration training which the pharmacists receive in pharmacology, the research team decided to exclude pharmacists from the current study.

Students had access to podcasts of seven pharmacology lectures which were delivered during the course. In order to gain a detailed insight into students' experiences of using podcasts sampling for this interview study was purposive in nature. Students were classified into three categories (non- or low users, medium users and high users) depending on how many of the podcasts they had used. This information was gained from tracking student use of the podcasts on WebCT (the virtual learning environment through which podcasts were made available) and documenting whether each student had clicked on the link to each of the individual podcasts. Four high users (used 6-7 podcasts), two medium users (used 3-5 podcasts) and one low user (used 0-2) of the podcasts agreed to take part in the interviews. The research team thought it would be helpful to use this tracking information to identify participants who may have different experiences with or opinions on using the podcasts.

All seven participants (23% of the total cohort) were female which was generally representative of the gender make-up of the cohort which was 83% female. Four participants worked in a hospital setting and three were community-based nurses. Ages ranged from 34-54 with a mean age of 43, which is representative of the age range of students enrolled on NMP courses at the University of Nottingham identified in previous research [8].

### Interview process

Participants took part in semi-structured interviews which lasted approximately 20-35 minutes. Interviews were conducted in a private interview room within the university or at the student's workplace, depending on their preference. An interview schedule was used to guide the interview conversation. However, this schedule was used in a flexible way to allow for unanticipated topics relevant to students experiences of using podcasts to emerge. The interview schedule topics focused on students' prior experience of using podcasts, their initial reactions to the introduction of pharmacology podcasts, reasons for use/non-use of podcasts, perceived advantages and disadvantages of podcasting for their learning and barriers and facilitators of podcasts use.

### Data Analysis

Data were analysed thematically using template analysis [32,33]. This approach was particularly appropriate as the researchers had some specific aspects of the students' experience of podcasts upon which they wished to focus their attention, i.e. student experiences of using the podcasts and barriers to, and facilitators of, podcast use. These specific aspects were used to guide the initial development of a coding template. However, emergent themes were also added to the coding template throughout the analysis process.

All interview transcripts were initially coded for emergent themes. A preliminary coding template was then developed. Codes were organised in a hierarchical manner such that the template contained a number of higher-order overarching themes which encompassed a variety of lower-order themes and sub-themes. This template was developed and amended through careful re-reading of all the interview transcripts and the application of successive drafts of the template to the full data set. This iterative process of amending the template and recoding the template continued until the authors were confident that the template allowed as full a description of the data as was achievable. The final coding template is given in Table 1.

**Table 1:** Final Coding Template

*First level code*	*Second level code*	*Third level code*
Use of podcasts	Reasons for use	Fuller comprehension
		Revision (General and exam)
		Missed class
		Specific learning needs/questions
	
	Barriers to use	Lack of awareness of technology
		Lack of access to technology
		Difficulties downloading to MP3
		Time restraints
	
	Facilitators of use	Family support
		I.T. assistance
		Ease of access

Impact on learning	Enhanced control over learning	Learning at own pace
		Gauging study needs using podcasts
		Portability increasing learning opportunities
	
	Adding value to course materials	Complementary learning tool
		Independent supplementary learning tool
	
	Building understanding	Building understanding of complex topic
		Access to extra information
		Repetition reinforcing knowledge
		Recovering lost information

### Ethical Approval

This project received ethical approval from the University of Nottingham Medical School Ethics Committee (Project Code M/5/2008).

## Results

The emergent themes from the template analysis are presented below. These themes will be discussed under two over-arching themes: use of podcasts and impact on learning. For the purposes of brevity when quoting participants, "..." is used to denote where an encouraging prompt from the interviewer (e.g. yes, yeah, emhem) has been excluded and "......" is used to denote where a greater section of text has been excluded. Any clarifying comments from the authors are included within parentheses in the quoted text. Letters A to G are used to refer to the responses from the different participants.

### 1. Use of Podcasts

The first overarching theme that is presented below is student use of the podcasts. Student use of podcasts is presented under the three emergent themes: reasons for, facilitators of, and barriers to podcast use. Facilitators and barriers with regards to podcast use will be discussed simultaneously, due to the highly related nature of these themes.

#### 1.1 *Reasons for use*

When students who had used the podcasts were asked why they did so, all participants described revisiting the lecture so as to get a fuller comprehension of what they felt was a complex subject as a main reason for use. They often felt that although they might have understood the lecture materials during the lecture, it was difficult to retain the information after the lecture had finished:

*A: You know you hear (lecturer's name) say it and it makes sense. It's so clear and it's so easy to understand.......and as you come away you think oh God you know what did she say or whatever and...it would become much less clear*.

Some students felt that the materials were so complex that it was difficult to grasp all the information presented during the lecture. Participant C described how the complexity of the content of the pharmacology lectures was similar to learning a foreign language. This student, as well as others felt that the podcasts were useful for recovering any information they did not grasp during the lecture:

C: I kind of thought actually this could be quite good because there's things that you miss because there's so much French and German coming at you.

Most participants, including high, medium and low users, utilised the podcasts for revision purposes. Some used the podcasts to reinforce existing knowledge in order to prepare for exams:

*B: I'm tending to go back to the first lectures because it was so long ago that they were done and......reinforce my knowledge, the existing knowledge I've got in order that I can pass the exam*.

Apart from generally revisiting the lecture and exam preparation, students mentioned using the podcasts to recap on certain areas which they were having trouble with or if they had specific questions. For example, participant C used podcasts during revision when she was struggling with some of the material and needed to clarify specific topics. She used podcasts to reassure herself that she had the correct understanding of how the mechanisms of action of certain drugs worked:

C: I got really confused about what drugs would do what...... so I referred back to that (podcast) just to try and reassure myself that I was right.

Only two participants (one low and one medium user), used the podcasts because they had missed some pharmacology sessions and they wanted to catch up on the missed material. For example, interviewee D decided to download the podcast for the first few sessions as she had missed what she felt were important foundation lectures:

*D: The sessions that I missed were in the first week so sort of the foundations of pharmacology which I really didn't want to miss*.

The fact that only a few students used podcasts to catch up on missed lectures may be due to the fact that attendance at lectures is a course requirement.

#### 1.2 *Barriers to, and facilitators of, podcast use*

Participants' experiences of barriers to, and facilitators of podcast use, mostly centred around technological issues. Technological problems were experienced by high, medium and low users alike. One barrier that students experienced was a lack of access to the technology needed to use the podcasts. For one participant, a slow internet connection hampered her ability to download the podcasts:

*D: I was trying at home and unfortunately they're still on dial-up connection at home...So it was taking too long to download so I wasn't able to sort of listen to the full lecture*.

Other students reported that a lack of ownership of an MP3 player limited their use of the podcasts.

A low level of familiarity with the technology around downloading and listening to podcasts hampered some participants' use of this learning method:

*B: Because I wasn't very technically minded it was difficult...and that may be why in the beginning I didn't access them*,

A significant problem for a number of students seemed to be related to transferring the podcast file from their computer to an MP3 player:

A: I think we did try it (downloading the podcast to an MP3 player)...but there was a problem with it and we didn't know how to overcome that problem.

Due to these problems with downloading the MP3 files onto an MP3 player, some participants found it easier to listen to the podcasts via WebCT rather than download the recordings to an MP3 player:

C: I found it easier just to open them actually on WebCT and I stayed on WebCT and just had them on the computer.

The fact that they needed help with the technology side of using the podcasts presented a potential barrier to use for some students. However, most students did report that they received help from others in order to facilitate their podcast use. Participant B, for example, felt she may not have used the podcasts if it had not been for the help she received help from her family in order to download the podcasts onto her MP3 player.

B: I probably wouldn't have used the podcasts had my family not been able to download them on to my iPod for me. I just didn't have that knowledge... but obviously other people in the family did so they helped me out with that basically.

For some students, receiving advice from I.T. personnel at the university facilitated their use of the podcasts:

*E: He told me to make it into a file and download it*.

Once some participants had succeeded in initially overcoming some of the technological problems they were having relating to using the podcasts, they found that the ease with which they could access and use the podcasts was a facilitator of their subsequent podcast use:

B: I've got all (the podcasts) that I can possibly download and I think if there were another ten on there......I would have downloaded them. You know now I know how to do it it's easy but it's not simple initially

A final barrier to podcast use mentioned by some students was the lack of time available to students to listen back to lectures. Busy work and family lives meant that some interviewees found it difficult to make time to listen to the podcasts:

*F: I just never got around to it.......It was quite difficult in that it was around Christmas and our exams were just in January......and that was busy*.

### 2. Impact on learning

The impact of podcast availability on students' learning will now be presented under four separate, but overlapping, sub-themes: enhanced control; gauging study needs; adding value to existing course materials; and building understanding.

#### 2.1 Enhanced control

Interviewees who were either medium or high users found that the availability of podcasts increased their control over their learning in a number of ways. In comparison to a lecture scenario, participants felt that by using the podcasts, they could manage the pace of the lecture delivery in a way that allowed them to stop and focus on areas which they may need more time to digest:

*F: I remember sitting in the lecture and thinking yep I understand that and you know but remember thinking but stop there because I need to just focus on that bit that I've just understood before you've gone on to that so...I knew that downloading it I'd be able to just listen and listen and listen again*.

The availability of podcasts which related to both basic principles of pharmacology as well as more complex subject topics, also allowed some students to control the pace at which they built up their comfort level with the subject. For example, participant B used the podcasts for revision purposes in an incremental way as her confidence in the subject increased. She did so by listening to the podcasts which contained more foundational information first before listening to podcasts relating to specific, more complex topics:

*B: I'm working on the basics at the minute and...... as that level of knowledge grows and I feel more comfortable with it...I add in another one (podcast)*.

Another way in which students gained enhanced control over their learning through using podcasts was related to the portability of the podcasts as learning tools. Some students felt that this portability enhanced their control over when and where they accessed the podcasts:

*E: I just found that it was another way of being able to learn without having to be sitting at a desk doing it...so you could get on with your life as well while you were learning*.

#### 2.2 Gauging Study Needs

Participants also used podcasts to gauge their understanding of certain topics and therefore modified their learning according to their needs. Often they would then replay specific sections of the podcasts in order to grasp the topics which they were finding more difficult to understand. Participant G, for example, was asked whether she listened to entire podcasts or listened to specific parts of the podcast and replied:

*G: It was as a whole originally but then what I did was if there was anything I wasn't clear on I went back into certain sections*.

Similarly, some participants used the podcasts to highlight areas which they wanted to retrieve further information on from other learning resources:

*F: I would listen straight through and then I'd make notes about certain things that I wanted to you know be more specific on and then go back to...look up wherever, computer, books, what have you and then go back to that*.

Therefore, podcasts had a positive impact on participants' level of control of their own learning as participants used podcasts as a marker of their understanding of the lecture materials and as an indicator of further study needs.

#### 2.3 Adding value to existing learning materials

All students discussed the value of podcasts as a very useful addition to the learning materials for their pharmacology course. As discussed in the previous section (2.2), students used podcasts in tandem with other more traditional learning resources (e.g. textbooks and lecture notes). Participants felt that the availability of podcasts as a supplementary learning tool was highly valuable for their learning as the podcasts complemented the other, more traditional resources available:

*C: I did find them (podcasts) useful especially when I started putting them together with the handout*.

As well as complementing existing learning resources, students found podcasts valuable as a supplementary learning tool which they could use independently. Interviewee E valued the extra study time that she was able to fit in due to the portability of the podcasts and the fact that she could use this independent of other learning materials outside of the home:

E: That extra work that I was doing I wouldn't have done...because I would be still having to do those things I was doing outside of the house...but it was like an extra learning opportunity

#### 2.4 Building Understanding

As discussed previously (see section 1.1), participants found the pharmacological content on the NMP course quite complex. In general, all participants found that the podcasts helped them to build their understanding of this difficult subject:

*C: Pharmacology is very new and very it's very complex. It's very high tech so...I think the podcasts really helped me in that*.

Interviewees suggested that the podcasts were particularly useful for pharmacology due to the scientific, fact-based nature of the subject. Some students felt that podcasting would not be as helpful for subjects that are not as fact-based as pharmacology.

D: I think perhaps (podcasts are) more helpful for pharmacology because you are dealing with scientific fact. Some of the other sessions were a bit more...open debate learning rather you know sort of putting your heads together rather than learning facts.

While not all participants ruled out the usefulness of podcasts in other areas of the prescribing course, most felt that podcasts had the most value in the pharmacology aspect of the course.

Participants mentioned some specific ways in which the podcasts helped them to understand the course materials. Some participants found that the extra information available on the podcasts was advantageous as the verbal explanation of the lecture materials allowed them to make more sense of the lecture material when revising. Participant B found that in comparison with other learning resources (books and lecture slides), the extra spoken explanations of the information on the lecture handouts in the podcasts enabled her to gain a greater understanding of the subject:

*B: They're good (books and lecture notes)...... but I think in order for them to be explained for me to think about it logically in my own head it's nice to have the podcasts there because you talk around that slide don't you when you're teaching*.

Some participants found aural learning to be a more useful method of assimilating the lecture content than reading:

*E: I'm much more likely to be taking it in listening to it than I am sitting at a table reading it*.

The ability to replay the lecture podcasts several times helped some students to assimilate the information required for a fuller comprehension of the subject and to reinforce their knowledge of the topic area:

G: When you actually sit there and listen to it again it actually makes a lot more sense and it sinks in better...and you can confirm everything that you've learned.

As discussed previously in section 1.1, participants found podcasts helpful for building their understanding as they were able to recover some bits of information that they would have otherwise forgotten from the lecture:

C: You can be in that lecture and you can think I'm really getting that. It's coming to me but then there's bits that would just drop out of your brain and then suddenly it would become a bit of a jigsaw again...So it was really good to be able to go back to the podcast and go that's what she said...now I remember.

## Discussion

The purpose of the current study was to gain an in-depth insight into NMP students' experiences of using podcasts as supplementary leaning tools in the pharmacology component of their course. In particular, the focus of the research was on identifying student reasons for accessing podcasts, their experiences of facilitators of, and barriers towards, podcast use and to identify how students used the podcast in their learning of pharmacology. Consistent with our previous study with this unique student population [8], students reported using the podcasts for a variety of reasons including revisiting the lecture for a fuller comprehension of the materials, revising, finding answers to specific questions, and to a lesser extent to catch up on missed lectures. This correlates well with data from both Copley [15] and Pilarski and colleagues [5] who reported that revision and general review of lectures were the main reasons for student usage of podcasts in marine science and molecular foundations of medicine respectively. While these authors used supplementary podcasts in different ways, i.e. using audio and video podcasts [15] or using podcasts which were linked to PowerPoint slides [5], it seems that the key reasons for use are similar. Relatively few students used podcasts for missed lectures but those who needed to do so found it helpful to have the podcast available.

Many students listened to podcasts via the computer rather than actually utilising the full portability of the technology by downloading to an MP3 player [3,8,18]. This trend is consistent with previous research [3,8,16]. Some researchers have suggested that this trend may be related to the older ages of students taking part in courses [8,18]. However, the undergraduate students in the study by Evans [3] were a homogenous younger age group, suggesting other explanations for this behaviour. The importance of the data presented in the current study is that it explores the first-hand experiences of students in relation to the use of podcasts, which have not previously been reported in the literature and may help unravel some of these anomalies.

Barriers to, and facilitators of podcast use were highly inter-related. Barriers to podcast use included a lack of access to appropriate technology, lack of awareness of technology and problems associated with downloading podcasts. Facilitators of podcast mainly consisted of help received from others, including family, friends and University staff, in terms of accessing the podcasts. The fact that students initially struggled to download podcasts onto MP3 players could be due to a combination of a lack of technological expertise by this group of students and the nature of the virtual learning environment currently being used [8]. It is clear then that providing early I.T. support should be an important consideration in terms of facilitating podcast use. Further technological advances in online learning environments may also make the process of downloading podcasts a more user-friendly experience. In the meantime, and in response to this data, we have moved the full lecture recordings to a site outside of WebCT (but which is accessed through WebCT) which has made downloading of the podcasts significantly easier. Anecdotally, this has increased portable use, with current students describing listening to podcasts while 'doing the ironing', 'laying the decking in the garden' and 'walking the dog'.

A further barrier to podcast use which emerged from this study was a reported lack of time available to listen back to the lecture recordings. This lack of time, whilst consistent with previous research on mature student learners, who tend to have multiple commitments (e.g. family, work etc.) outside of their college work [28,29], may arise from the student tendency to listen to podcasts via the computer. Students may have needed more time to sit and listen to the podcasts via the computer than they perhaps would have needed if they had downloaded the podcasts to an MP3 player for mobile use. Improving the ease of downloading podcasts to MP3 players, as has been done with the most recent cohort of students, and thus enhancing the portability of learning may act to overcome this barrier.

The second important component of this study was the investigation of how students felt the availability of podcasts impacted upon their learning. A significant finding emerging from the analysis was the fact that high and medium users felt that the podcasts allowed them to increase their level of control over their studies in a variety of ways, as they could: better pace their engagement with the lecture materials; use the podcasts in a portable manner to increase their opportunities to engage with the lecture materials; and listen back to the lecture materials to assess their level of understanding and tailor their further study accordingly.

Non-medical prescribing students felt that podcasts were a valuable addition to the learning resources on the course as both a learning tool which could be used independently or in conjunction with other learning resources. They also describe the podcasts as helpful in building their understanding of a complex subject by allowing them to access extra verbal information from the lecture which they would not normally have, by allowing them to replay the lecture materials as often as required for fuller comprehension of the topic and by allowing them to recover information from the lecture which they otherwise may have forgotten. The ability to revisit the lecture on multiple occasions may help alleviate students' anxiety regarding missing lecture content while taking notes. Indeed, the knowledge that lecture recordings are available may allow students to focus better on the lecture material itself rather than making notes, listening and trying to develop an understanding simultaneously. This type of divided attention has been shown to have a substantially detrimental effect on recall and recognition performance [34]. The ability to give full attention in the lecture would increase recall and recognition performance and reduce the likelihood of misinformation being learned.

Moreover, the addition of an aural component to the repertoire of available supplementary learning tools should not be underestimated. Indeed, the addition of a studio recording aural component to a web-based lecture course in surgery resulted in an increased exam performance [35]. This increased performance may be the result of students accessing the aural component whilst looking through the slides. This bimodal format of information presentation (audio-visual) has been demonstrated to result in better recall compared to unimodal formats [reviewed in [36]], possibly because bimodal items are encoded twice in the working memory.

Whilst the move to downloading lecture recordings to MP3 players could mean that students do not sit at a computer for prolonged periods of time while also studying lecture slides, the audio-visual format of encoding information would not necessarily be lost. The increased portability of the podcasts would enhance student opportunities to utilise lecture recordings and printed slides together by making this exercise much more flexible in terms of where and when it could be done.

The preference expressed by some students to listen to verbal information via the podcasts rather than using other study techniques, such as reading books or notes, suggests a preference for an aural learning style. This type of preference has been previously noted in undergraduate physiology students, with those expressing this preference performing better overall [37]. Perhaps more importantly, the addition of these aural learning tools enhances students' opportunity to access different learning styles or modes, which has been suggested to be important for deep learning [38].

The limitations of this study are a reflection of its specificity; NMP students are a unique group with specific needs. Consequently, the results of this study may not be fully applicable to all students studying all subjects. Other, more discursive, subject areas for example, may not lend themselves as well as pharmacology to the use of podcasting. Similarly, other student groups may not utilise podcasts in the same manner as the NMP students interviewed in this study. While the availability of lecture recordings as podcasts has not resulted in increased absenteeism with this specific group of students [8], the same may not be true of other student groups. The results of this study do however provide a number of useful insights into student learning, some of which are likely to be applicable to other student groups, and could therefore provide encouragement for teaching staff to move towards embracing this technology for its impact on student learning rather than simply for the technology itself.

## Conclusions

The aim of this study was to investigate NMP students' experiences of using pharmacology podcasts. Students reported barriers to podcast use that were generally technical in nature. These issues may be negated as technology advances but can also be overcome through careful planning and the provision of early support. The benefits of the use of podcasts were multiple in nature and most importantly included giving students greater control over their own learning. Students felt that the ability to repeatedly access the aural component of the lecture was essential for the development of a complete understanding of complex lecture material. In summary, students advocated the use of podcasts to support and enhance their learning processes. These data suggest that teaching staff should strongly consider the use of podcasts to augment student learning.

## Competing interests

The authors declare that they have no competing interests.

## Authors' contributions

JSL and DB conceived of, designed the study and obtained the funding. JSL and OM designed the interview schedule. OM conducted and transcribed the interviews. OM and JSL analysed the data. OM and JSL drafted the manuscript. All authors have read and approved the final manuscript.

## Pre-publication history

The pre-publication history for this paper can be accessed here:

http://www.biomedcentral.com/1472-6920/11/2/prepub
